# 6-Shogaol from *Zingiber officinale* Induces Cell Cycle Arrest via Suppression of c-Myc Protein Expression and Promotes Apoptosis in Human B-Cell Lymphoma

**DOI:** 10.3390/ijms27104168

**Published:** 2026-05-07

**Authors:** Sirinya Moakmamern, Lapamas Rueankham, Natsima Viriyaadhammaa, Wenxian Yin, Toyonobu Usuki, Suwit Duangmano, Yupanun Wutti-In, Sawitree Chiampanichayakul, Nutjeera Intasai, Siriporn Okonogi, Singkome Tima, Songyot Anuchapreeda

**Affiliations:** 1 Department of Medical Technology, Faculty of Associated Medical Sciences, Chiang Mai University, Chiang Mai 50200, Thailand; sirinya_moakm@cmu.ac.th (S.M.); lapamas.rk96@gmail.com (L.R.); natsima.v@cmu.ac.th (N.V.); yinwenxian@wmu.edu.cn (W.Y.); suwit.du@cmu.ac.th (S.D.); yupanun.wuttiin@cmu.ac.th (Y.W.-I.); sawitree.chiampa@cmu.ac.th (S.C.); nutjeera.in@cmu.ac.th (N.I.); singkome.tima@cmu.ac.th (S.T.); 2 Cancer Research Unit of Associated Medical Sciences (AMS CRU), Faculty of Associated Medical Sciences, Chiang Mai University, Chiang Mai 50200, Thailand; 3 Department of Materials and Life Sciences, Faculty of Science and Technology, Sophia University, Tokyo 102-8554, Japan; t-usuki@sophia.ac.jp; 4 Center of Excellence in Pharmaceutical Nanotechnology, Chiang Mai University, Chiang Mai 50200, Thailand; okng2000@gmail.com; 5 Department of Pharmaceutical Sciences, Faculty of Pharmacy, Chiang Mai University, Chiang Mai 50200, Thailand

**Keywords:** 6-shogaol, ginger, *Zingiber officinale*, cancer, lymphoma, c-Myc, apoptosis, bioactive food compounds, nutraceutical

## Abstract

Lymphoma is a hematological malignancy and a major non-communicable disease characterized by the uncontrolled proliferation of lymphoid cells, frequently associated with dysregulation of the cellular myelocytomatosis (c-Myc) oncogenic pathway. In this study, we investigated the anti-lymphoma potential of bioactive compounds derived from edible plants in the Zingiberaceae family, including fingerroot (*Boesenbergia rotunda*), turmeric (*Curcuma longa*), white turmeric (*Curcuma mangga*), zedoary (*Curcuma zedoaria*), and ginger (*Zingiber officinale*). Crude extracts from these traditionally consumed medicinal food plants were evaluated for cytotoxic effects against human B-cell lymphoma cell lines (Raji and Daudi) and compared with normal peripheral blood mononuclear cells (PBMCs). Among the tested extracts, ginger and turmeric exhibited selective cytotoxicity toward lymphoma cells. Ginger was selected for further investigation, and subsequent analyses identified 6-shogaol as the principal active compound. 6-Shogaol significantly suppressed total and phosphorylated c-Myc protein expression, induced dose-dependent apoptosis, and caused cell cycle arrest in lymphoma cells. Network pharmacology and pathway enrichment analyses suggested the modulation of multiple oncogenic signaling pathways, particularly the PI3K/Akt/c-Myc and MAPK signaling. These findings indicate that 6-shogaol exerts anti-lymphoma activity through the coordinated modulation of oncogenic and apoptotic pathways. However, as this study is limited to in vitro and computational analyses, further in vivo validation is required. Overall, 6-shogaol represents a promising food-derived lead compound for the development of novel therapeutic strategies against lymphoma.

## 1. Introduction

Lymphoma is a hematolymphoid malignancy originating from the lymphatic system, a key component of the immune network composed primarily of B cells, T cells, and natural killer (NK) cells. Globally, lymphoma represents a significant health burden, accounting for approximately 3–4% of all cancers, with increasing incidence and mortality observed in many regions. Non-Hodgkin’s lymphoma (NHL) is the most prevalent subtype, comprising about 90% of cases and exhibiting substantial clinical and histological diversity, whereas Hodgkin’s lymphoma accounts for the remaining 10% [1,2,3]. Despite advances in treatment, lymphoma remains a major cause of cancer-related morbidity and mortality worldwide, with marked geographic variation in incidence and outcomes [2]. Among children, it is the third-most common cancer after leukemia and tumors of the brain and nervous system. In Thailand, epidemiological data from 2016 to 2018 indicate that NHL was among the most prevalent malignancies, ranking fifth in men (6.6%) and ninth in women (5.2%) [4]. Most lymphomas (85–95%) are of B-cell origin [5], with diffuse large B-cell lymphoma (DLBCL, NOS) being the predominant subtype, accounting for 58.1% of NHL cases in the Thai population [6]. These findings are consistent with global trends and further highlight the need for more effective and selective therapeutic strategies for lymphoma. B lymphocytes, derived from hematopoietic stem cells in the bone marrow, play a central role in immune defense through antibody production. Their development is tightly regulated by the cellular myelocytomatosis (c-Myc) proto-oncogene, which governs cell proliferation, growth, and apoptosis [7]. Located on chromosome 8q24, c-Myc is frequently involved in chromosomal translocations, particularly t(8;14)(q24;q32), leading to abnormal overexpression through juxtaposition with immunoglobulin gene enhancers. This dysregulation is a hallmark of Burkitt’s lymphoma and is further influenced by signaling pathways including PI3K, AKT, and mTOR [8].

Recent advances in lymphoma therapy have focused on targeted agents and immunotherapies, including BTK inhibitors, bispecific antibodies, and CAR T-cell therapies, which have significantly improved clinical outcomes in patients with B-cell lymphomas [9,10,11]. However, challenges such as treatment resistance, toxicity, and limited accessibility remain [11]. Therefore, the identification of novel bioactive compounds with multi-target mechanisms and improved safety profiles remains important. In this context, food-derived compounds such as 6-shogaol may offer complementary therapeutic potential.

Current standard-of-care treatments for lymphoma include combination chemotherapy regimens such as ABVD (doxorubicin, bleomycin, vinblastine, and dacarbazine) for Hodgkin’s lymphoma and R-CHOP (rituximab, cyclophosphamide, doxorubicin, vincristine, and prednisone) for non-Hodgkin lymphoma. Despite their clinical effectiveness, these regimens are associated with nonspecific cytotoxicity toward rapidly dividing normal cells, frequently leading to severe adverse effects, including alopecia, nausea, vomiting, and diarrhea [12]. Chemotherapy, often combined with hematopoietic stem cell transplantation (HSCT), therefore remains limited by toxicity, highlighting the need for safer therapeutic alternatives. These limitations have prompted increasing interest in alternative therapeutic approaches, particularly plant-derived compounds with anticancer potential. Natural products such as shikonin have been reported to suppress lymphoma cell proliferation by inducing caspase-dependent apoptosis and inhibiting the c-Myc and PI3K/Akt/mTOR signaling pathways [13]. Similarly, curcuminoids exhibit diverse biological activities and relatively low toxicity; for example, curcumin has been shown to suppress tumor growth in Burkitt’s lymphoma xenograft models [14,15], although its direct effect on c-Myc signaling remains unclear.

The Zingiberaceae family, widely consumed as both traditional medicinal plants and dietary components, has attracted considerable attention for its anticancer properties, including the inhibition of cell proliferation and induction of apoptosis. As food-derived bioactive sources, Zingiberaceae constituents represent promising therapeutic candidates for non-communicable diseases, including cancer. Previous studies have demonstrated the anti-leukemic potential of compounds such as curcumin, which downregulates WT1 expression via PKCα signaling; bisdemethoxycurcumin, which inhibits leukemic stem cells [16]; and 6-gingerol, which induces ROS-mediated apoptosis in myeloid cells [17,18]. Notably, 6-shogaol has demonstrated stronger growth-inhibitory effects than 6-gingerol in several cancer cell lines [19]. In addition, zerumbone has been reported to suppress the proliferation of Raji lymphoma cells by modulating apoptosis-related gene expression, underscoring its therapeutic potential in lymphoma [20]. Despite these promising findings, studies investigating the modulation of c-Myc, a critical oncogenic driver, by Zingiberaceae constituents in lymphoma models remain limited. Therefore, the present study initially evaluated the cytotoxic potential of crude extracts and major pure compounds derived from five Zingiberaceae species: fingerroot (*Boesenbergia rotunda*), turmeric (*Curcuma longa*), white turmeric (*Curcuma mangga*), zedoary (*Curcuma zedoaria*), and ginger (*Zingiber officinale*). The most potent extract was subsequently selected for mechanistic investigations focusing on lymphoma cell proliferation and apoptosis.

Given the limited availability of therapies that selectively target malignant lymphoma cells while sparing normal cells, this study aimed to evaluate the cytotoxic and selective anti-lymphoma activities of extracts from five Zingiberaceae species against Raji and Daudi cells, using normal peripheral blood mononuclear cells (PBMCs) as controls. Raji and Daudi are human B-cell lymphoma cell lines derived from Burkitt’s lymphoma, a highly aggressive and well-established c-Myc-driven malignancy. This model was selected because dysregulation of the c-Myc oncogene is a defining feature of Burkitt’s lymphoma, making it particularly suitable for investigating compounds that target c-Myc-associated signaling pathways. Following initial screening, the most active extract and its major bioactive compound were further investigated for their ability to suppress c-Myc expression and modulate downstream signaling pathways involved in cell cycle arrest and apoptosis, particularly the PI3K/Akt/c-Myc and MAPK signaling pathways. These findings may provide a scientific basis for the development of food-derived, plant-based therapeutic candidates with improved selectivity for lymphoma treatment.

Although 6-shogaol has been widely reported to exhibit anticancer activity in leukemia and various solid tumors, its effects in lymphoma, particularly in relation to c-Myc regulation, remain largely unexplored. To date, limited evidence exists demonstrating the ability of 6-shogaol to suppress c-Myc expression and induce apoptosis in Burkitt’s lymphoma models. Given the central role of c-Myc in lymphoma pathogenesis, investigating its modulation by food-derived bioactive compounds such as 6-shogaol is of considerable interest. Therefore, the present study aimed to evaluate the cytotoxic and mechanistic effects of 6-shogaol in lymphoma, with particular focus on c-Myc suppression and apoptosis induction.

## 2. Results

### 2.1. Cytotoxicity of Crude Extracts from Zingiberaceae Plants on Lymphoma Cell Lines

In this study, the crude ethanolic extracts of five Zingiberaceae plants were evaluated for their cytotoxic effects against Raji and Daudi lymphoma cell lines. All extracts exhibited varying levels of cytotoxicity. Among them, turmeric showed the strongest activity in both cell lines, particularly in Daudi cells, with IC_50_ values of 13.21 ± 1.44 and 7.68 ± 0.56 µg/mL in Raji and Daudi cells, respectively (Table 1). Ginger also demonstrated potent cytotoxicity, with IC_50_ values of 18.46 ± 4.23 and 17.07 ± 1.98 µg/mL, respectively. The selectivity index (SI), defined as the ratio of IC_50_ in normal cells to those in cancer cells, serves as an indicator of the therapeutic window and safety profile of a compound [21,22,23]. All crude extracts showed SI values greater than 1, indicating greater selectivity toward lymphoma cells than normal PBMCs (Table 1). Ginger exhibited favorable SI values of 3.83 and 4.14 in Raji and Daudi cells, respectively, and was therefore selected for further investigation. Based on previous literature [24,25], 6-gingerol and 6-shogaol were identified as the major active constituents of ginger and were subsequently examined for their biological activities.

### 2.2. Cytotoxicity of Major Compounds from Ginger on Lymphoma Cell Lines and Normal PBMCs

The two major active compounds from ginger, 6-gingerol and 6-shogaol, were evaluated for their cytotoxic effects against Raji and Daudi lymphoma cell lines, as well as normal PBMCs. 6-Shogaol exhibited the strongest cytotoxic activity, with IC_50_ values of 3.31 ± 0.29 and 2.45 ± 0.16 µg/mL in Raji and Daudi cells, respectively. In contrast, 6-gingerol showed considerably lower cytotoxicity in both lymphoma cell lines, with IC_50_ values of 96.11 ± 3.16 and 53.79 ± 15.40 µg/mL, respectively. However, 6-shogaol also exhibited cytotoxicity toward normal PBMCs, with an IC_50_ value of 10.02 ± 0.96 µg/mL, whereas 6-gingerol showed minimal toxicity (IC_50_ > 100 µg/mL) (Table 2). The SI values of 6-gingerol, 6-shogaol, and doxorubicin were all greater than 1, indicating greater selectivity toward lymphoma cells than PBMCs (Table 2). Nevertheless, because the IC_50_ values of 6-gingerol in both lymphoma cell lines were relatively high, it was excluded from subsequent experiments. To further validate the selection of ginger over turmeric, a direct comparison between 6-shogaol and curcumin was performed. The results demonstrated that 6-shogaol exhibited greater cytotoxic potency than curcumin in both Raji and Daudi lymphoma cell lines, as indicated by lower IC_50_ values (Table 2). Specifically, the IC_50_ values of 6-shogaol in Raji and Daudi cells were lower than those of curcumin, which were 4.81 ± 0.99 and 3.59 ± 0.53 µg/mL, respectively. These findings provide additional support for selecting 6-shogaol as the lead bioactive compound for further mechanistic investigation. However, curcumin exhibited slightly higher selectivity indices than 6-shogaol, suggesting somewhat greater selectivity toward lymphoma cells. Despite this, the stronger cytotoxic potency of 6-shogaol, together with its relatively underexplored role in lymphoma, supports its selection as the lead bioactive compound for further mechanistic investigation.

### 2.3. Effect of 6-Shogaol on Cell Cycle Arrest in Raji and Daudi Cells

**Figure 1:**
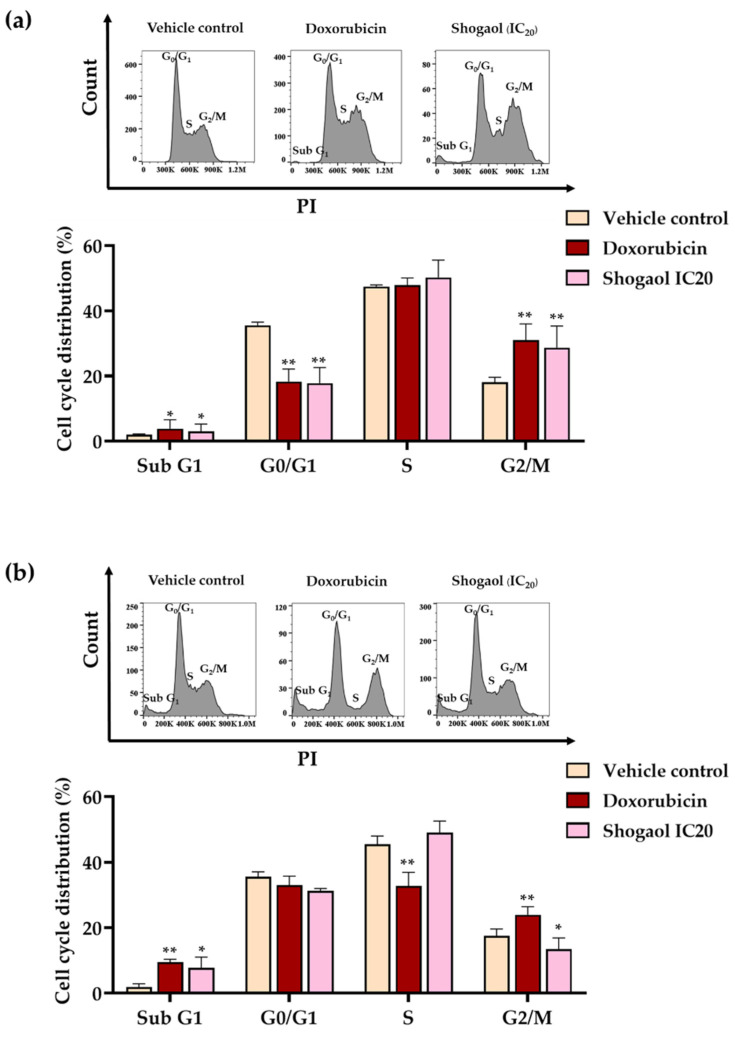
Effect of 6-shogaol on cell cycle distribution in Raji and Daudi cells. (**a**) Raji cells at a density of 1 × 10^5^ cells/mL were treated with 6-shogaol at the IC_20_ concentration for 48 h. (**b**) Daudi cells at a density of 2 × 10^5^ cells/mL were treated with 6-shogaol at the IC_20_ concentration for 48 h. Doxorubicin was used as a positive control. Data are expressed as mean ± SD from three independent experiments. Asterisks (*) denote significant differences compared with the vehicle control (* *p* < 0.05, ** *p* < 0.01).

The MTT assay, which measures cytotoxicity, cannot distinguish whether the reduction in cell viability results from the inhibition of cell proliferation or the induction of cell death. To clarify this, cell cycle analysis was performed. Raji and Daudi cells were treated with 6-shogaol at concentrations corresponding to their IC_20_ values, specifically 1.95 µg/mL for Raji cells and 1.75 µg/mL for Daudi cells, as determined by the MTT assay. The IC_20_ concentration was selected to minimize excessive cytotoxicity and allow accurate evaluation of cell cycle distribution. Higher concentrations, such as the IC_50_, may induce substantial apoptosis, thereby confounding the interpretation of cell cycle phase alterations. After 48 h of treatment, cell cycle distributions across the G0/G1, S, and G2/M phases were assessed by PI staining followed by flow cytometric analysis. In Raji cells, 6-shogaol induced a significant accumulation of cells in the G2/M phase compared with the vehicle controls (*p* < 0.05), showing a pattern similar to that of the chemotherapeutic drug doxorubicin (Figure 1a). Conversely, in Daudi cells, 6-shogaol did not induce phase-specific cell cycle arrest but markedly increased the percentage of cells in the sub-G1 phase, indicating apoptotic cell death (Figure 1b). The positive control, doxorubicin, induced cell cycle arrest at the G2/M phase in Raji cells and at the G0/G1 phase in Daudi cells.

### 2.4. Effects of 6-Shogaol on c-Myc and Phosphorylated c-Myc Protein Expressions in Raji and Daudi Cells

**Figure 2:**
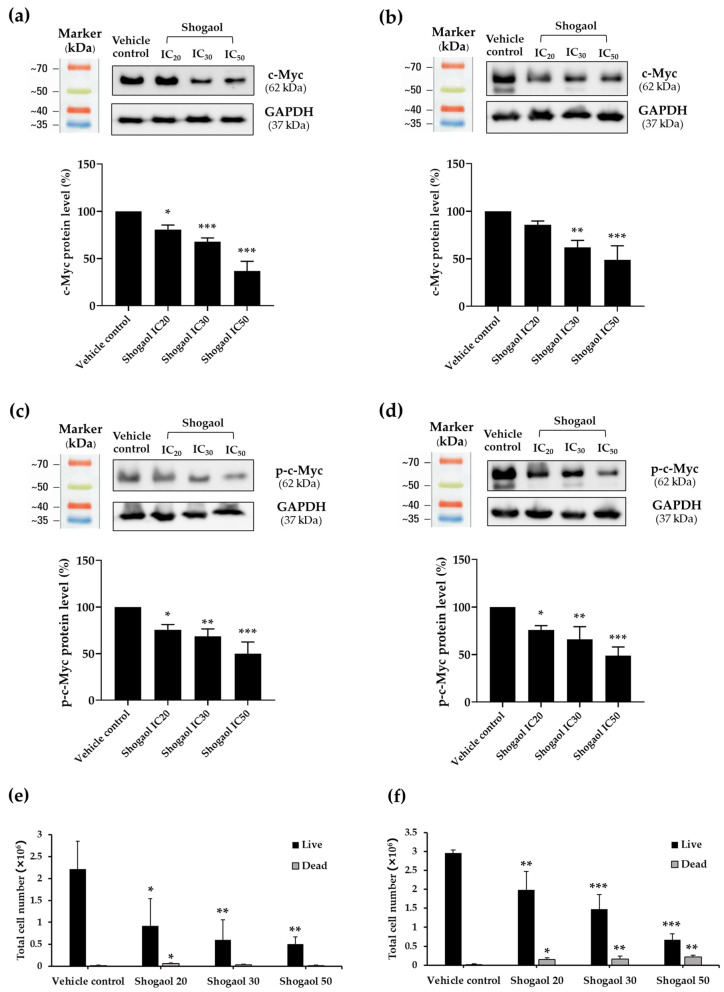
Effects of 6-shogaol on c-Myc and p-c-Myc protein levels and total cell numbers in Raji and Daudi cells. c-Myc and p-c-Myc protein levels were evaluated by Western blotting, and total cell numbers were determined using the trypan blue exclusion assay. Panels (**a**,**b**) show c-Myc protein levels in Raji and Daudi cells, respectively, after 48 h of treatment with 6-shogaol at the IC_20_, IC_30_, and IC_50_ concentrations, quantified by densitometric analysis of Western blots. Panels (**c**,**d**) depict p-c-Myc protein levels under the same conditions in Raji and Daudi cells, respectively. Panels (**e**,**f**) illustrate total cell numbers measured by trypan blue exclusion in Raji and Daudi cells following 48 h of treatment with 6-shogaol. Data are represented as the mean ± SD from three independent experiments. Protein levels were normalized to GAPDH as a loading control. Asterisks indicate significant differences compared with the vehicle controls (* *p* < 0.05, ** *p* < 0.01, *** *p* < 0.001).

To determine whether 6-shogaol affects c-Myc signaling, both total c-Myc and its phosphorylated form (p-c-Myc) were examined following treatment. Raji and Daudi lymphoma cells were exposed to 6-shogaol at concentrations corresponding to the IC_20_, IC_30_, and IC_50_ for 48 h. The IC_20_, IC_30_, and IC_50_ values for Raji cells were 1.95, 2.38, and 3.31 µg/mL, respectively, while those for Daudi cells were 1.75, 1.99, and 2.45 µg/mL, respectively. Protein levels of c-Myc and p-c-Myc were analyzed by Western blotting. The results revealed a clear dose-dependent suppression of both c-Myc and p-c-Myc. Specifically, in Raji cells, 6-shogaol reduced c-Myc expression by 19.30, 32.29, and 63.30% (Figure 2a), while p-c-Myc levels decreased by 24.38, 31.65, and 50.23% (Figure 2c), respectively. A similar trend was observed in Daudi cells, with c-Myc levels decreasing by 14.20, 38.16, and 51.34%, and p-c-Myc levels decreasing by 24.27, 34.19, and 51.29% (Figure 2b,d), corresponding to increasing concentrations of 6-shogaol. These findings demonstrate that 6-shogaol effectively suppresses c-Myc expression and activation in lymphoma cells in a dose-dependent manner, highlighting its therapeutic potential for targeting dysregulated cell proliferation.

### 2.5. Effect of 6-Shogaol on Total Cell Number in Raji and Daudi Cells

Treatment with 6-shogaol resulted in a pronounced reduction in total cell number, accompanied by a significant increase in cell death, effects that were particularly evident in Daudi cells (Figure 2e,f). Following 48 h of exposure to 6-shogaol at IC_20_, IC_30_, and IC_50_ concentrations, cell viability assessed by trypan blue exclusion revealed a clear dose-dependent decrease in the number of viable cells in both lymphoma lines. In Raji cells, total cell numbers decreased by approximately 58.23, 72.70, and 77.19% with increasing doses of 6-shogaol (Figure 2e). A similar trend was observed in Daudi cells, with reductions of 32.77, 50.17, and 77.48%, respectively. Notably, 6-shogaol also triggered a dose-dependent increase in the percentage of non-viable, trypan blue-positive Daudi cells, rising by 7.01, 10.45, and 24.81% (Figure 2f). These results demonstrate that 6-shogaol exerts a strong antiproliferative effect on lymphoma cells while simultaneously promoting cell death, particularly in Daudi cells. The trypan blue exclusion assay further supports that the reduced cell number is associated with both inhibited proliferation and increased cell death.

### 2.6. Network Analysis of 6-Shogaol for Predicting Targets Against Lymphoma

**Figure 3:**
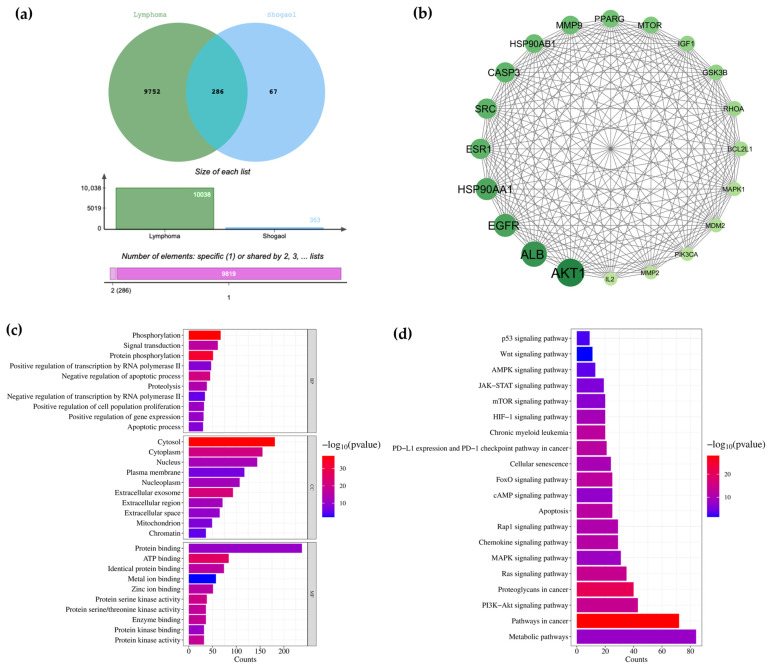
Network pharmacology-based prediction of 6-shogaol targets in lymphoma. Panel (**a**) shows a Venn diagram illustrating the overlap among identified gene sets obtained from the bioinformatic analysis of the lymphoma–6-shogaol target network. The green circle represents gene targets exclusively associated with lymphoma, whereas the blue circle represents gene targets specific to 6-shogaol. The overlapping region indicates gene targets shared between lymphoma and 6-shogaol. Panel (**b**) presents the protein–protein interaction (PPI) network of the top 20 key gene targets of 6-shogaol against lymphoma. Panel (**c**) shows gene ontology (GO) enrichment analysis of the overlapping gene targets, including the top 10 enriched terms in biological processes (BP), cellular components (CC), and molecular functions (MF). Panel (**d**) illustrates KEGG pathway enrichment analysis showing the top 20 signaling pathways associated with the potential gene targets of 6-shogaol in lymphoma.

PharmMapper and SwissTargetprediction identified 353 potential targets for 6-shogaol. Lymphoma-associated genes were retrieved from two human gene databases, DisGeNET and GeneCards, resulting in 10,038 candidate gene targets. The identified gene targets were categorized into three groups: (1) lymphoma-related genes, (2) 6-shogaol-targeted genes, and (3) overlapping genes between lymphoma and 6-shogaol targets. As shown in Figure 3a, 286 overlapping gene targets were identified between lymphoma and 6-shogaol. To investigate the molecular mechanisms underlying the anti-lymphoma activity of 6-shogaol, protein–protein interaction (PPI) networks were constructed using the STRING database and visualized with Cytoscape. The top 20 key gene targets were selected based on node degree (Figure 3b and Supplementary Table S1). Functional enrichment analysis of the 286 overlapping genes was performed using DAVID. The results suggested that 6-shogaol may modulate phosphorylation, signal transduction, and apoptosis-related processes in cellular compartments such as the cytosol, cytoplasm, nucleus, and plasma membrane. These processes were associated with molecular functions including protein binding, ATP binding, and metal ion binding, which may collectively contribute to the suppression of lymphoma progression (Figure 3c). KEGG pathway analysis revealed enrichment of key pathways, including metabolic pathways, pathways in cancer, PI3K-Akt signaling, MAPK signaling, and apoptosis (Figure 3d). Taken together, these findings suggest that 6-shogaol may exert its anti-lymphoma effects partly through the modulation of Akt-related signaling within the PI3K-Akt pathway, thereby inhibiting lymphoma progression.

### 2.7. Molecular Docking Analysis of 6-Shogaol Against Key Lymphoma-Related Targets

**Figure 4:**
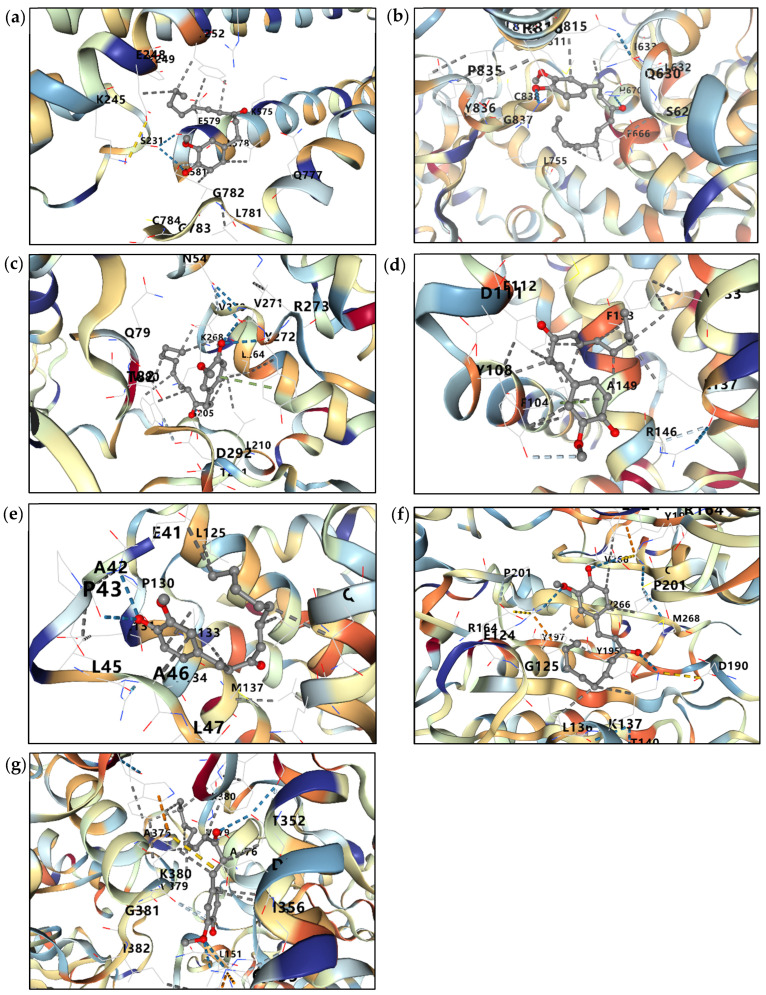
Molecular docking analysis of 6-shogaol against key lymphoma-related target proteins. Representative docking poses of 6-shogaol with (**a**) c-Myc, (**b**) PI3K, (**c**) Akt, (**d**) Bcl2, (**e**) Bax, (**f**) Casp3, and (**g**) Casp9 are shown. The predicted binding conformations illustrate the interactions of 6-shogaol within the active or binding sites of the selected proteins. Binding energies (kcal/mol) were calculated by molecular docking analysis and are summarized in Table 3. Among the tested targets, the strongest binding affinity was observed for Casp9 (−8.4 kcal/mol), followed by Akt (−8.2 kcal/mol), supporting the potential involvement of apoptosis-related and PI3K/Akt signaling pathways in the anti-lymphoma effects of 6-shogaol. The red and gray structures represent overlaid ligand conformations of 6-shogaol within the binding site, while the target protein is shown as a ribbon model.

To further explore the potential molecular interactions underlying the anti-lymphoma effects of 6-shogaol, molecular docking analysis was performed against key proteins involved in cell proliferation, survival signaling, and apoptosis, including c-Myc, PI3K, Akt, Bcl2, Bax, caspase 3 (Casp3), and caspase 9 (Casp9). The docking results demonstrated that 6-shogaol exhibited favorable binding affinities toward all selected targets, with binding energies ranging from −6.5 to −8.4 kcal/mol (Table 3 and Figure 4). Among these, the strongest binding was observed with Casp9 (−8.4 kcal/mol), followed by Akt (−8.2 kcal/mol). Moderate binding affinities were also detected for Bax (−7.1 kcal/mol), PI3K (−7.0 kcal/mol), Casp3 (−7.0 kcal/mol), Bcl2 (−6.6 kcal/mol), and c-Myc (−6.5 kcal/mol). These findings suggest that 6-shogaol may exert its anti-lymphoma activity through multi-target interactions, particularly by modulating PI3K/Akt-associated survival signaling and apoptosis-related proteins. The observed interactions with Casp3, Casp9, Bax, and Bcl2 further support the pro-apoptotic effects of 6-shogaol observed in the experimental assays.

### 2.8. Effects of 6-Shogaol on Cleaved-Casp3 Expression in Raji and Daudi Cells by Western Blotting

**Figure 5:**
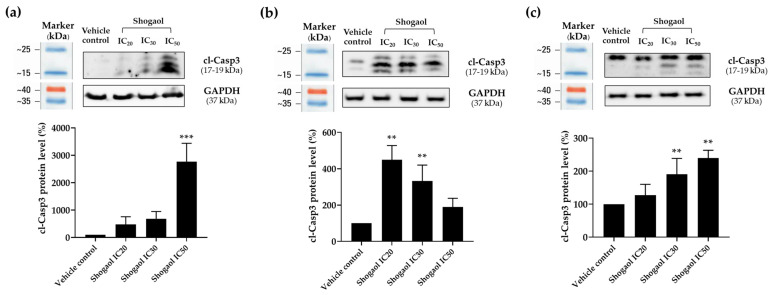
Effects of 6-shogaol on cl-Casp3 protein expression in Raji and Daudi cells. Cleaved caspase-3 (cl-Casp3) protein levels were evaluated by Western blotting. Panel (**a**) shows cl-Casp3 protein levels in Raji cells after 48 h of treatment with 6-shogaol at IC_20_, IC_30_, and IC_50_ concentrations, quantified by densitometric analysis. Panel (**b**) shows cl-Casp3 protein levels in Daudi cells after 48 h of treatment under the same conditions. Panel (**c**) shows cl-Casp3 protein levels in Daudi cells after 24 h of treatment with 6-shogaol at IC_20_, IC_30_, and IC_50_ concentrations. Data are expressed as mean ± SD from three independent experiments. Protein levels were normalized to GAPDH as a loading control. Asterisks indicate significant differences compared with vehicle controls (** *p* < 0.01, *** *p* < 0.001).

Cleaved caspase-3 (cl-Casp3) is a key executioner protease in apoptosis and serves as a definitive marker of apoptosis activation. In this study, the pro-apoptotic effects of 6-shogaol were examined in Raji and Daudi lymphoma cell lines by measuring cl-Casp3 protein levels after treatment. Cells were exposed to 6-shogaol at IC_20_, IC_30_, and IC_50_ concentrations for 48 h. Western blot analysis revealed a dose-dependent increase in cl-Casp3 expression, which was particularly pronounced in Raji cells after 48 h. At the IC_50_ concentration, 6-shogaol induced a 27.66-fold increase in cl-Casp3 levels compared with the vehicle control (Figure 5a). In Daudi cells, cl-Casp3 expression also increased following 48 h of treatment but displayed a different pattern, peaking at a 4.46-fold increase at IC_20_ and decreasing to a 2.36-fold increase at IC_50_ (Figure 5b). Notably, at 24 h, cl-Casp3 levels in Daudi cells showed a consistent dose-dependent increase of 1.27-, 1.90-, and 2.40-fold at IC_20_, IC_30_, and IC_50_, respectively (Figure 5c), suggesting transient activation of Casp3. Collectively, these findings demonstrate that 6-shogaol activates the Casp3-mediated apoptotic pathway in lymphoma cell lines.

### 2.9. Effects of 6-Shogaol on Apoptosis in Raji and Daudi Cells by Flow Cytometric Analysis

**Figure 6:**
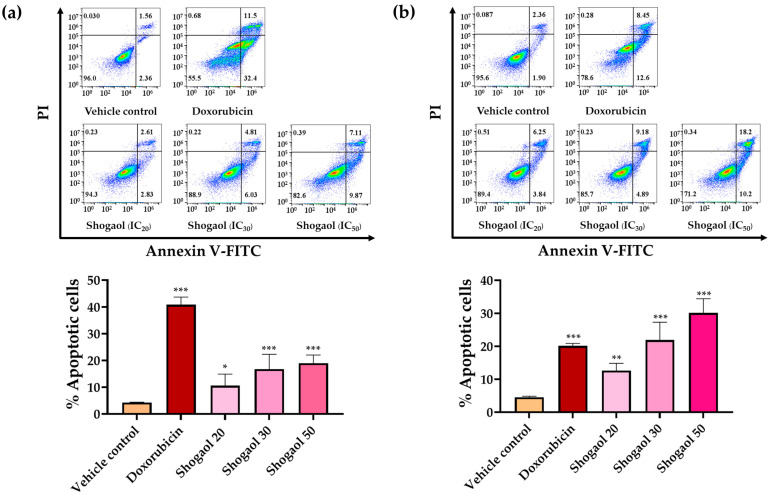
Effects of 6-shogaol on apoptosis in Raji and Daudi cells. Apoptosis was evaluated by Annexin V-FITC/PI staining followed by flow cytometry. Apoptotic cells were identified based on Annexin V-FITC and PI staining. Panel (**a**) shows Raji cells (1.0 × 10^5^ cells/mL) treated with 6-shogaol at IC_20_, IC_30_, and IC_50_ concentrations for 48 h. Panel (**b**) shows Daudi cells (2.0 × 10^5^ cells/mL) treated with 6-shogaol at IC_20_, IC_30_, and IC_50_ concentrations for 48 h. Representative flow cytometry dot plots show the distribution of cell populations (**top**), and quantitative data are presented as the mean ± SD from three independent experiments (**bottom**). Asterisks indicate significant differences compared with vehicle controls (* *p* < 0.05, ** *p* < 0.01, *** *p* < 0.001) (**bottom**).

To further confirm the pro-apoptotic effects of 6-shogaol, Annexin V-FITC/propidium iodide (PI) dual staining was performed, followed by flow cytometric analysis. This method distinguishes early apoptotic cells (Annexin V^+^/PI^−^), late apoptotic or necrotic cells (Annexin V^+^/PI^+^), and viable cells (Annexin V^−^/PI^−^). Raji cells (1.0 × 10^5^ cells/mL) and Daudi cells (2.0 × 10^5^ cells/mL) were treated with IC_20_, IC_30_, and IC_50_ concentrations of 6-shogaol for 48 h, with doxorubicin serving as a positive control. The analysis revealed a dose-dependent increase in the proportion of apoptotic cells in both cell lines following treatment. In Raji cells, 6-shogaol induced apoptosis in a concentration-dependent manner, with apoptotic rates of 10.58 ± 4.34, 16.79 ± 5.56 and 19.03 ± 3.06% at IC_20_, IC_30_, and IC_50_ concentrations, respectively (Figure 6a). Conversely, Daudi cells exhibited higher apoptotic rates upon 6-shogaol treatment, with percentages of 12.64 ± 2.24, 21.94 ± 5.41, and 30.17 ± 4.24% at IC_20_, IC_30_, and IC_50_ concentrations, respectively, after 48 h (Figure 6b). Notably, this increase was consistent with the elevated cl-Casp3 expression observed in Section 2.8. Together, these findings corroborate the biochemical evidence from cl-Casp3 analysis and demonstrate that 6-shogaol effectively induces apoptosis in lymphoma cells.

## 3. Discussion

Members of the Zingiberaceae family, widely consumed as culinary ingredients and traditional medicinal foods, have recently attracted significant interest because of their potential to alleviate chemotherapy-induced side effects and enhance lymphoma treatment efficacy. Previous studies have shown that food-derived bioactive compounds such as curcumin and zerumbone exert anti-lymphoma effects by inhibiting the NF-κB pathway and promoting mitochondrial-mediated apoptosis through the upregulation of pro-apoptotic proteins and suppression of c-Myc expression in Burkitt’s lymphoma cells. Zerumbone, a sesquiterpene isolated from wild ginger (*Zingiber zerumbet*), has demonstrated the ability to suppress the proliferation of Raji lymphoma cells by inducing late-stage apoptosis and modulating apoptosis-related genes, specifically increasing Bax while decreasing Bcl-2 and c-Myc, highlighting its promise as a therapeutic agent against Burkitt’s lymphoma. In the present study, crude extracts from five edible Zingiberaceae species, including fingerroot (*Boesenbergia rotunda*), turmeric (*Curcuma longa*), white turmeric (*Curcuma mangga*), zedoary (*Curcuma zedoaria*), and ginger (*Zingiber officinale*), were evaluated against Raji and Daudi lymphoma cell lines. All extracts demonstrated cytotoxicity to varying degrees, with turmeric exhibiting the strongest activity. Comparative analysis revealed that 6-shogaol exhibited greater cytotoxic potency than curcumin in lymphoma cell lines, as reflected by lower IC_50_ values. Although curcumin demonstrated slightly higher selectivity indices, its anticancer mechanisms have been extensively characterized in lymphoma and other cancers. In contrast, 6-shogaol represents a less explored bioactive compound in lymphoma, particularly in relation to c-Myc regulation. Therefore, the present study focused on 6-shogaol to provide novel mechanistic insights into its anti-lymphoma activity.

The selectivity index (SI) is commonly used to evaluate the therapeutic potential of anticancer compounds, with values greater than 3 generally indicating selective cytotoxicity toward cancer cells. In the study, 6-shogaol demonstrated SI values exceeding this threshold, suggesting favorable selectivity. However, as cytotoxicity was assessed only in PBMCs, further evaluation in additional normal cell types is required.

Ginger (*Zingiber officinale*), a widely consumed spice and functional food ingredient from the Zingiberaceae family, has long been used both as a culinary component and traditional medicine. Its rhizome is a rich source of bioactive dietary phytochemicals, particularly phenolic compounds and flavonoids [26]. Among these, 6-gingerol and 6-shogaol are the principal active constituents, with 6-gingerol being the most abundant fresh rhizome component and 6-shogaol generated through dehydration during thermal processing. Both compounds exhibit antioxidant, anti-inflammatory, and anticancer properties [27]. Previous studies have demonstrated that 6-gingerol exerts antiproliferative effects in multiple cancer types; for example, it inhibits HeLa cell growth by inducing G0/G1 cell cycle arrest through downregulation of cyclins A and D1 and promotes apoptosis via caspase activation and mTOR inhibition. Additionally, both 6-gingerol and 6-shogaol suppress proliferation in human prostate cancer cells by downregulating multidrug resistance-associated protein 1 (MRP1) and glutathione S-transferase (GSTπ) [28,29]. Notably, 6-shogaol demonstrates stronger anticancer activity than 6-gingerol, with greater growth inhibition observed in lung (H1299) and colon (HCT-116) cancer cell lines [19], and has also been reported to induce reactive oxygen species (ROS) production and increase tumor suppressor p53 expression in liver cancer cells [30]. Collectively, these studies indicate complementary but distinct anticancer mechanisms for 6-gingerol and 6-shogaol, with the latter generally showing superior potency in inhibiting cancer cell growth and triggering apoptosis. Consistent with these observations, our study demonstrated that 6-shogaol exhibited markedly stronger cytotoxicity than 6-gingerol against Raji and Daudi lymphoma cells, with IC_50_ values of 3.31 ± 0.29 and 2.45 ± 0.16 µg/mL, respectively. Cell cycle analysis further revealed that 6-shogaol at the IC_20_ concentration for 48 h induced G2/M phase arrest in Raji cells but did not induce phase-specific arrest in Daudi cells. Instead, it significantly increased the sub-G1 population, particularly in Daudi cells, indicating enhanced apoptotic cell death. This differential response likely reflects phenotypic differences between the two cell lines: Raji cells harbor mutated p53, resulting in impaired DNA damage response and S phase or G2/M arrest, whereas Daudi cells express wild-type p53 and may therefore undergo apoptosis more readily [31].

As a major food-derived bioactive compound from ginger, 6-shogaol inhibited the expression of both c-Myc and p-c-Myc in a clear dose-dependent manner, showing a similar trend in both Raji and Daudi cell lines. Treatment with the IC_50_ concentration for 48 h suppressed more than 50% of c-Myc and p-c-Myc expression, indicating substantial inhibition of this critical oncogenic signaling axis. These findings are consistent with earlier reports demonstrating that 6-shogaol suppresses oncogenic pathways such as STAT3 and NF-κB and downregulates downstream survival-related proteins, including cyclin D1, survivin, and c-Myc, thereby reducing cell survival more effectively than 6-gingerol in prostate cancer cells [32]. In agreement with this mechanism, 6-shogaol also decreased cell viability in both lymphoma cell lines in a dose-dependent manner by more than 70%, while inducing cell death in less than 25% of cells, suggesting that inhibition of proliferation is a major contributing mechanism alongside apoptosis induction. Collectively, these findings further support the potential of 6-shogaol as a promising food-derived anti-lymphoma bioactive compound with possible therapeutic relevance for cancer-related non-communicable diseases. Importantly, while previous studies have demonstrated the anticancer effects of 6-shogaol in leukemia and solid tumors, evidence regarding its role in lymphoma remains limited. In particular, direct evidence linking 6-shogaol to c-Myc suppression in lymphoma has not been clearly established. In this study, we demonstrate for the first time that 6-shogaol significantly suppresses both total and p-c-Myc expression in Burkitt’s lymphoma cells, accompanied by the induction of apoptosis. These findings provide novel mechanistic insight into the anti-lymphoma activity of this food-derived bioactive compound.

Network pharmacology and molecular docking analyses further strengthened the proposed anti-lymphoma mechanism of 6-shogaol as a food-derived multi-target bioactive compound. Bioinformatic analysis identified 286 overlapping targets between lymphoma-associated genes and 6-shogaol-related targets. Among these, PPI network analysis highlighted several high-confidence hub genes, including Akt1, EGFR, and Casp3, based on degree centrality. Importantly, these hub genes are closely associated with the experimental findings observed in this study. Casp3, a key executioner of apoptosis, is consistent with the increased cl-Casp3 expression detected in lymphoma cells following 6-shogaol treatment. Similarly, Akt1 is a central regulator of the PI3K/Akt signaling pathway, supporting our observation that 6-shogaol modulates survival signaling in lymphoma cells. GO and KEGG enrichment analyses further demonstrated significant involvement in phosphorylation, signal transduction, apoptosis, and the PI3K-Akt and MAPK signaling pathways, which are known to regulate cell proliferation, survival, and programmed cell death in B-cell lymphoma. Collectively, these results demonstrate strong concordance between computational predictions and experimental validation, supporting the multi-target mechanism of 6-shogaol in lymphoma. Notably, molecular docking demonstrated favorable binding affinities of 6-shogaol toward several key proteins involved in these pathways, particularly Akt (−8.2 kcal/mol), PI3K (−7.0 kcal/mol), Casp3 (−7.0 kcal/mol), and Casp9 (−8.4 kcal/mol). The predicted interactions with apoptosis-related proteins are consistent with our experimental findings, which showed increased cl-Casp3 expression and elevated apoptotic cell populations in both Raji and Daudi cells following 6-shogaol treatment. Furthermore, the moderate binding affinity toward c-Myc (−6.5 kcal/mol), together with the marked suppression of total and p-c-Myc protein levels, suggests that 6-shogaol may regulate c-Myc-associated signaling through both direct and indirect mechanisms. Collectively, these findings support a multi-target mode of action in which 6-shogaol suppresses lymphoma cell growth through modulation of PI3K/Akt/c-Myc survival signaling while potentially promoting apoptosis-related pathways. However, the involvement of specific upstream regulators of intrinsic apoptosis requires further experimental validation.

Casp3 was selected as the primary apoptosis marker in this study because it functions as a key executioner caspase and provides direct evidence of apoptosis induction. In contrast, Casp9 acts as an upstream initiator of the intrinsic apoptotic pathway. Although molecular docking analysis suggested potential interactions between 6-shogaol and Casp9, this finding remains predictive and was not experimentally validated; therefore, the involvement of Casp9 in 6-shogaol-induced apoptosis requires further investigation. In the present study, 6-shogaol induced dose-dependent apoptosis in Raji and Daudi lymphoma cells, as evidenced by increased cl-Casp3 expression and elevated Annexin V-positive populations. In Raji cells, 6-shogaol triggered a 27.66-fold increase in cl-Casp3 levels at the IC_50_ concentration. In Daudi cells, Casp3 activation peaked at the IC_20_ concentration and declined at higher doses, suggesting transient activation or engagement of alternative cell death pathways. This interpretation is supported by time-course analysis showing dose-dependent increases in cl-Casp3 expression at 24 h in Daudi cells. Both cell lines exhibited a dose-dependent increase in apoptotic populations, with Daudi cells showing higher apoptosis rates (up to 30.17%) compared with Raji cells (up to 19.03%). The increased apoptotic cell populations were highly consistent with cl-Casp3 activation, confirming Casp3 as a key mediator of 6-shogaol-induced programmed cell death. Molecular docking and network pharmacology analyses further suggested potential interactions of 6-shogaol with apoptosis-related proteins, including Casp9 and Bax; however, these findings are predictive and require experimental validation. Because upstream regulators of intrinsic apoptosis, such as Bax, Bcl-2, and Casp9, were not experimentally assessed in this study, the involvement of mitochondrial-mediated intrinsic apoptosis remains tentative. Further studies examining mitochondrial membrane potential and upstream apoptotic regulators are needed to clarify whether 6-shogaol preferentially activates intrinsic or extrinsic apoptotic pathways. Collectively, these findings support the pro-apoptotic activity of 6-shogaol in lymphoma cells and highlight its potential as a food-derived bioactive compound with therapeutic relevance.

Although molecular docking analysis revealed stronger predicted binding affinities of 6-shogaol toward apoptosis-related proteins such as Casp9 and signaling molecules such as AKT1, these results should be interpreted as supportive evidence of multi-target interactions rather than as indicators of primary target specificity. In this study, c-Myc was selected as the central focus due to its well-established role as a key oncogenic driver in Burkitt’s lymphoma. The observed suppression of c-Myc and p-c-Myc protein expression provides direct experimental evidence supporting this mechanism. Meanwhile, the predicted interactions with AKT1 and Casp9 are consistent with the modulation of survival and apoptosis pathways observed in our experimental assays, suggesting that 6-shogaol exerts its anti-lymphoma activity through coordinated multi-target effects.

In this study, doxorubicin was used as a clinically relevant positive control for cytotoxicity and apoptosis induction because it is widely used in lymphoma chemotherapy. Although doxorubicin primarily acts through DNA intercalation and topoisomerase II inhibition, its inclusion provided a reference for evaluating the anti-lymphoma cytotoxic and pro-apoptotic effects of 6-shogaol. However, because doxorubicin is not a pathway-specific inhibitor of c-Myc or PI3K/Akt signaling, future studies incorporating c-Myc- or PI3K/Akt-targeting agents would provide additional mechanistic validation.

Despite the promising in vitro anti-lymphoma activity of 6-shogaol, its clinical translation may be limited by pharmacokinetic challenges, including low stability and poor oral bioavailability. Previous studies have suggested that the plasma concentrations achievable through dietary consumption are likely lower than the effective concentrations observed in vitro. Therefore, the direct extrapolation of these findings to clinical settings should be interpreted with caution. To overcome these limitations, advanced delivery strategies, such as nanoparticle-based formulations, liposomal encapsulation, or other targeted delivery systems, may enhance the stability, bioavailability, and tumor-specific accumulation of 6-shogaol. In addition, combination therapy with existing chemotherapeutic agents or targeted inhibitors may further improve therapeutic efficacy while reducing the required dose. These approaches may provide a more feasible strategy for translating the anticancer potential of 6-shogaol into clinical applications.

Overall, this study highlights 6-shogaol, a major food-derived bioactive compound from ginger, as a potent anti-lymphoma and pro-apoptotic agent that acts through suppression of the oncogenic c-Myc signaling axis and modulation of PI3K-Akt-associated survival pathways, while potentially involving apoptosis-related mechanisms. These mechanistic insights further underscore the therapeutic and nutraceutical potential of Zingiberaceae-derived compounds as complementary strategies for the prevention and treatment of lymphoma and other cancer-related non-communicable diseases.

## 4. Materials and Methods

### 4.1. Crude Extracts of Five Zingiberaceae Plants

In June 2019, fresh rhizomes of *Boesenbergia rotunda*, *Curcuma longa*, *Curcuma mangga*, *Curcuma zedoaria*, and *Zingiber officinale* were collected from a local garden in Chiang Mai, Thailand. These edible and medicinal Zingiberaceae species are widely used in traditional Thai cuisine and herbal medicine and are not classified as endangered plants. The plant materials were authenticated by Wannaree Charoensup from Chiang Mai University, and voucher specimens were deposited at the Herbarium of the Faculty of Pharmacy, Chiang Mai University (deposition numbers: 009724 for *B. rotunda*, 023356 for *C. longa*, 0023362 for *C. mangga*, 0023369 for *C. zedoaria*, and 0023361 for *Z. officinale*).

Ethanolic extracts were prepared from fresh rhizomes using the maceration method. Briefly, each rhizome was dried, powdered, and macerated in 95% ethanol at a ratio of 1:3 (*w*/*v*) for 48 h. The mixture was filtered through cloth, and the residue was subjected to three additional maceration cycles under identical conditions. The combined filtrates were concentrated to dryness using a rotary evaporator, and the resulting crude extracts were stored in light-protected containers at −20 °C until use. Standard compounds, including 6-gingerol and 6-shogaol, were purchased from Sigma-Aldrich (St. Louis, MO, USA).

### 4.2. Cell Culture Conditions of Lymphoma Cell Lines

Raji and Daudi are human B-cell lymphoblastoid suspension cell lines derived from patients with Burkitt’s lymphoma. Raji cells carry the t(8;14) chromosomal translocation, which results in the juxtaposition of the *Myc* gene to the immunoglobulin heavy-chain (IgH) enhancer, and contain latent Epstein–Barr virus (EBV) in a non-productive state. In contrast, Daudi cells harbor the complete EBV genome and express mRNA of the proto-oncogene BCL2. The Raji cell line (EP-CL-0189) was obtained from Elabscience^®^ (Houston, TX, USA), whereas the Daudi cell line (CCL-213) was purchased from the American Type Culture Collection (ATCC) (Manassas, VA, USA). Both cell lines were cultured in RPMI-1640 medium (Roswell Park Memorial Institute, Thermo Fisher Scientific, Waltham, MA, USA) supplemented with 10% fetal bovine serum, 2 mM L-glutamine, 100 units/mL penicillin, and 100 µg/mL streptomycin (Invitrogen^®^, Carlsbad, CA, USA). The cells were maintained at 37 °C in humidified incubators containing 5% CO_2_. The growth curves of both cell lines are shown in Supplementary Figure S1.

### 4.3. Determination of Total Cell Number by Trypan Blue Exclusion Assay

Cell viability and total cell number were determined using the trypan blue exclusion assay. Live cells with intact plasma membranes exclude trypan blue dye and therefore remain unstained, whereas dead cells with compromised membranes absorb the dye and appear blue-stained. Briefly, 10 µL of cell suspension was mixed with 10 µL of 0.2% trypan blue solution, and viable (unstained) and non-viable (blue-stained) cells were counted using a hemocytometer. The percentage of viable cells and total cell number were subsequently calculated based on the average cell counts obtained from independent fields.

### 4.4. Cytotoxicity Assay of Crude Extracts and Major Compounds from Zingiberaceae on Lymphoma Cell Lines

The cytotoxic effects of Zingiberaceae extracts on Raji and Daudi cells were evaluated using the MTT assay (3-(4,5-dimethylthiazol-2-yl)-2,5-diphenyl tetrazolium bromide) in three independent experiments. After harvesting and washing three times with PBS (pH 7.4), Raji and Daudi were seeded at densities of 1.0 × 10^4^ cells/mL and 2.0 × 10^4^ cells/mL, respectively, based on the cell growth curve (Figure S1), into 96-well plates and incubated at 37 °C with 5% CO_2_ for 24 h. Following incubation, the cells were treated with crude extracts from Zingiberaceae plants at concentrations ranging from 3.125 to 100 µg/mL and further incubated for 48 h. Subsequently, 15 µL of MTT solution (5.0 g/L) was added to each well, and the plates were incubated for 4 h. The resulting formazan crystals formed by metabolically active cells were dissolved in DMSO, and absorbance was measured at 578 nm using a microplate reader (Metertech, Nankang, Taipei, Taiwan), with 630 nm serving as the reference wavelength. Cell viability was calculated using the following equation:

(1) $$\begin{array}{cc} \%\;\mathrm{Cell}\;\mathrm{viability}\;= & \frac{\mathrm{Mean}\;\mathrm{absorbance}\;\mathrm{in}\;\mathrm{test}\;\mathrm{well}}{\mathrm{Mean}\;\mathrm{absorbance}\;\mathrm{in}\;\mathrm{vehicle}\;\mathrm{control}\;\mathrm{well}} \end{array}\;\times \;100$$

The mean cell viability percentages obtained from three independent experiments at each concentration were used to generate dose–response curves. The inhibitory concentrations causing 20% (IC_20_) and 50% (IC_50_) reductions in cell growth were defined as the concentrations that decreased cell viability by 20% and 50%, respectively, compared to vehicle controls. Extracts and major pure compounds exhibiting low IC_50_ values, indicative of strong cytotoxic activity, were selected for subsequent assays in Raji and Daudi cells, together with comparative evaluation in normal peripheral blood mononuclear cells (PBMCs). DMSO (0.8%) was used as the vehicle control.

The selectivity index (SI) of active compounds and chemotherapeutic drugs was calculated according to the following equation.

(2) $$\mathrm{Selectivity}\;\mathrm{index}\;(\mathrm{SI})=\frac{{\mathrm{IC}}_{50\;N}}{{\mathrm{IC}}_{50\;C}},$$

where IC_50 N_ represents the IC_50_ value in normal cells (PBMCs), and IC_50 C_ represents the IC_50_ value in cancer cells treated with the same compounds.

### 4.5. Cytotoxicity of Pure Compounds from Zingiberaceae on PBMCs

#### 4.5.1. Preparation of PBMCs by Ficoll–Hypaque Density Gradient Centrifugation

PBMCs were isolated from whole blood using Ficoll–Hypaque density gradient centrifugation, which separates blood components based on density, allowing PBMCs to be separated from granulocytes and red blood cells (RBCs). Blood samples (10–20 mL) were collected from at least five healthy volunteers. The use of human PBMCs in this study was approved by the Human Research Ethics Committee of the Faculty of Associated Medical Sciences, Chiang Mai University (Approval No. 307/2024; approved on 3 July 2024). Written informed consent was obtained from all participants prior to sample collection. All participants were fully informed about the objectives and procedures of the study, and all experimental procedures were conducted in accordance with relevant ethical guidelines and regulations. Each sample was diluted 1:1 with sterile PBS to reduce viscosity, carefully layered onto Ficoll–Hypaque solution, and centrifuged at 400× *g* for 30 min at room temperature. The PBMC layer was carefully collected and washed twice with PBS by centrifugation at 2000 rpm for 10 min. Total cell number and cell viability were determined using the trypan blue exclusion assay.

#### 4.5.2. Cytotoxicity Effects of Major Pure Compounds from Candidate Zingiberaceae Plants

PBMCs isolated by Ficoll–Hypaque density gradient centrifugation were washed three times with PBS (pH 7.4) and adjusted to a density of 1.0 × 10^7^ cells/mL in complete RPMI-1640 medium supplemented with 10% FBS. The cells were seeded into 96-well plates and incubated at 37 °C in a humidified atmosphere containing 5% CO_2_ for 24 h. Subsequently, the cells were treated with the major pure compounds from candidate Zingiberaceae plants at concentrations ranging from 3.125 to 100 µg/mL and cultured for 48 h. Following treatment, 15 µL of MTT solution was added to each well and incubated for 4 h. The resulting formazan crystals were dissolved in DMSO, and absorbance was measured at 578 nm with a reference wavelength of 630 nm using a microplate reader.

### 4.6. Cell Cycle Arrest Analysis of Candidate Zingiberaceae Extracts and Major Pure Compounds in Lymphoma Cell Lines

Raji and Daudi cells were prepared at densities of 1.0 × 10^5^ and 2.0 × 10^5^ cells/mL, respectively, in complete RPMI-1640 medium supplemented with 0.5% FBS and incubated overnight at 37 °C in a humidified atmosphere containing 5% CO_2_ to induce serum starvation. Subsequently, the cells were treated with candidate Zingiberaceae extracts and their major pure compounds at concentrations equivalent to their IC_20_ values and further incubated under the same conditions for 48 h. Doxorubicin was used as a positive control and applied in the same manner. After treatment, the cells were collected, washed three times with cold PBS, pH 7.4, and transferred into microcentrifuge tubes. The cells were then fixed with ice-cold 70% ethanol in PBS, pH 7.4 and incubated at 4 °C in the dark for 30 min. Subsequently, the samples were centrifuged, the supernatants were discarded, and the cell pellets were stained with a propidium iodide (PI) working solution containing PI (20 µg/mL), EDTA (2 mM), Triton X-100 (0.1% *v*/*v*), and RNase A (8 µg/mL). Fluorescence intensity was measured by flow cytometry, and the data were analyzed using FlowJo V10 software.

### 4.7. Western Blot Analysis of c-Myc and Cleaved-Caspase-3 Protein Expression

Protein expression following 48 h of treatment with candidate pure compounds was analyzed by Western blotting. Both treated and untreated cells were lysed using a lysis buffer containing protease inhibitors, and protein concentrations were determined using the Pierce™ BCA Protein Assay Kit (ThermoFisher Scientific, Waltham, MA, USA). Equal amounts of protein (30 µg per sample) were separated on 12% SDS-PAGE gels and transferred onto PVDF membranes. After blocking with 5% skim milk in PBS, pH 7.4 for 2 h at room temperature, the membranes were incubated with primary antibodies against c-Myc and phospho-c-Myc (Ser62) (Cell Signaling Technology, Danvers, MA, USA) at a dilution of 1:1000, cleaved-Caspase-3 (cl-Casp3) (Cell Signaling Technology, Danvers, MA, USA) at 1:1000, and GAPDH (MilliporeSigma, Burlington, MA, USA) at 1:16,000 for 2 h with gentle agitation. Membranes initially probed for c-Myc were stripped using Restore™ PLUS Western Blot Stripping Buffer (Thermo Fisher Scientific, Waltham, MA, USA) before reprobing for phospho-c-Myc (p-c-Myc). Both proteins have an approximate molecular weight of 62 kDa. After washing, the membranes were incubated with HRP-conjugated goat anti-rabbit IgG secondary antibody (Promega, Madison, WI, USA) at a dilution of 1:20,000 for 1 h. Protein bands were detected using Luminata™ Forte Western HRP Substrate (Merck, Rahway, NJ, USA) and visualized using a ChemiDoc MP Imaging System (Bio-Rad, Hercules, CA, USA). Band intensities were quantified using Image Lab Software, version 6.1 (Bio-Rad, Hercules, CA, USA) and normalized to GAPDH as a loading control.

### 4.8. Network Pharmacology and Bioinformatic Analysis

To investigate the potential molecular mechanisms underlying the anti-lymphoma activity of 6-shogaol, a network pharmacology approach was employed. Potential targets of 6-shogaol were predicted using PharmMapper and SwissTargetPrediction. Lymphoma-associated genes were retrieved from the GeneCards (www.genecards.org) and DisGeNET databases. The overlapping gene targets between 6-shogaol-related targets and lymphoma-associated genes were identified and subsequently subjected to functional enrichment analysis.

Gene Ontology (GO) term classification and KEGG pathway enrichment analyses were performed using the DAVID platform (https://davidbioinformatics.nih.gov/ (accessed on 1 November 2025)). GO analysis classified the overlapping genes into three categories: Biological Process (BP), Cellular Component (CC), and Molecular Function (MF). KEGG pathway analysis was conducted to identify significantly enriched signaling pathways associated with lymphoma progression, apoptosis, and cell survival. The top 20 key gene targets were selected based on degree centrality values within the PPI network, with higher degree values indicating greater importance in network connectivity. The enrichment results were graphically visualized using SRplot (https://www.bioinformatics.com.cn/srplot (accessed on 1 November 2025)), and significantly enriched terms were presented as −log10 (*p*-value).

Protein–protein interaction (PPI) network analysis was performed using STRING version 12.0 (https://string-db.org/). The network data were visualized and analyzed for topological features using Cytoscape version 3.10.3 to identify major hub genes potentially involved in the anti-lymphoma mechanism of 6-shogaol.

Finally, molecular docking analysis was performed using CB-Dock2 to evaluate the interactions between 6-shogaol (retrieved from PubChem, https://pubchem.ncbi.nlm.nih.gov) and key lymphoma-related target proteins. The three-dimensional structure of 6-shogaol was retrieved from PubChem, while the crystal structures of target proteins were obtained from the Protein Data Bank, including c-Myc (PDB ID: 1NKP), AKT1 (PDB ID: 8UW7), PIK3CA (PDB ID: 8EXL), BCL2 (PDB ID: 6GL8), BAX (PDB ID: 4S0O), Casp 3 (PDB ID: 3DEI), and Casp 9 (PDB ID: 1JXQ). Potential binding cavities were identified using the curvature-based cavity detection algorithm implemented in CB-Dock2, and affinities were calculated as Vina scores (kcal/mol). The docking poses with the lowest binding energy for each target were selected as the representative binding conformations and subsequently visualized using PyMOL, version 3.0.4.

### 4.9. Investigation of Apoptosis Induction by Candidate Zingiberaceae Extracts and Major Pure Compounds in Lymphoma Cell Lines

Apoptosis induced by candidate Zingiberaceae extracts and their major pure compounds was evaluated by flow cytometry using the Annexin V-FITC Apoptosis Detection Kit (BD Biosciences, San Jose, CA, USA). Raji and Daudi cells were prepared at densities of 1.0 × 10^5^ and 2.0 × 10^5^ cells/mL, respectively, in RPMI-1640 medium supplemented with 10% FBS, and treated with plant extracts or pure compounds at concentrations ranging from IC_20_ to IC_50_. The cells were incubated for 48 h at 37 °C in a humidified atmosphere containing 5% CO_2_. Doxorubicin was used as a positive control. After treatment, the cells were collected, washed three times with cold PBS, pH 7.4, counted, and resuspended in Annexin V binding buffer at a density of 1 × 10^6^ cells/mL. Aliquots containing 1 × 10^5^ cells were stained with 5 µL Annexin V-FITC and propidium iodide (PI), gently mixed, and incubated in the dark for 15 min at 25 °C. The samples were then diluted with binding buffer and analyzed by flow cytometry within 1 h. Data were processed using FlowJo V10 software (BD Biosciences, San Jose, CA, USA), with unstained and single-stained controls used to establish gating parameters. The percentage of apoptotic cells was determined by subtracting the apoptosis rate of untreated controls from that of treated samples.

### 4.10. Statistical Analysis

Data are presented as the mean ± standard deviation (SD) from three independent experiments performed in triplicate. Statistical comparisons between groups were carried out using one-way analysis of variance (ANOVA). Differences were considered statistically significant at *p* < 0.05, *p* < 0.01, and *p* < 0.001.

## 5. Conclusions

**Figure 7:**
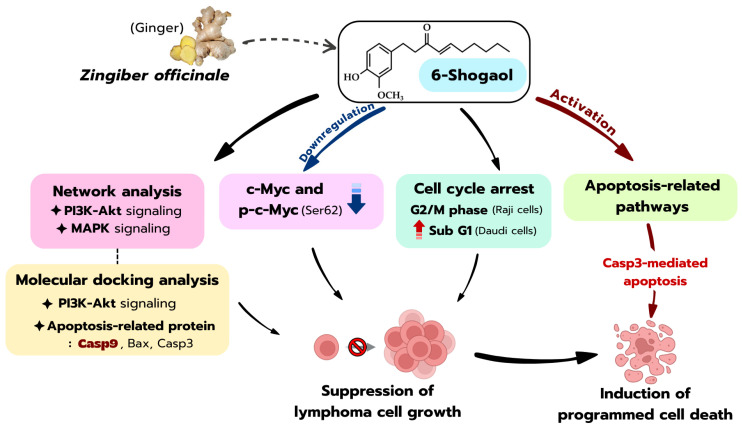
Proposed mechanisms underlying the anti-lymphoma effects of 6-shogaol derived from ginger (*Zingiber officinale*). 6-Shogaol exerts anti-proliferative and pro-apoptotic effects in Raji and Daudi lymphoma cells through coordinated modulation of multiple signaling pathways. It downregulates c-Myc and p-c-Myc (Ser62), while network analysis suggests the involvement of PI3K-Akt and MAPK signaling pathways. In addition, molecular docking analysis supports potential interactions with apoptosis-related proteins, particularly Casp9, Bax, and Casp3, although these findings are predictive in nature. These molecular alterations lead to cell cycle arrest in the G2/M phase in Raji cells and an increase in the sub-G1 population in Daudi cells, indicating apoptotic cell death. This is followed by activation of apoptosis-associated pathways, including Casp3-mediated apoptosis. Collectively, these coordinated mechanisms suppress lymphoma cell proliferation and promote programmed cell death.

This study demonstrates that 6-shogaol, a major food-derived bioactive compound from ginger (*Zingiber officinale*), exerts potent dose-dependent anti-lymphoma activity against Raji and Daudi lymphoma cell lines. Mechanistically, 6-shogaol inhibited lymphoma cell growth through multiple coordinated pathways (Figure 7), including suppression of c-Myc and p-c-Myc protein expression, modulation of PI3K/Akt- and MAPK-associated survival signaling, induction of cell cycle arrest, and induction of Casp3-mediated apoptosis. Network pharmacology and molecular docking analyses further supported its multi-target mechanism, highlighting key interactions with Akt, PI3K, and apoptosis-related proteins. However, it should be noted that the present findings are based on in vitro experiments and computational analyses. Therefore, 6-shogaol should be considered a promising lead compound rather than a confirmed therapeutic or nutraceutical agent. Future studies should focus on in vivo validation, pharmacokinetic optimization, and the development of effective delivery systems to further evaluate its clinical potential.

## Figures and Tables

**Table 1:** IC_50_ values and selectivity indices of the five Zingiberaceae plants in lymphoma cell lines and normal PBMCs.

Zingiberaceae Plant	IC_50_ Value (μg/mL) (Mean ± SD)			Selectivity Index (SI)	
	Raji	Daudi	PBMCs	Raji	Daudi
Boesenbergin (*Boesenbergia rotunda*)	25.89 ± 1.24	18.52 ± 0.84	39.83 ± 2.27 ^#^	1.54	2.15
Turmeric (*Curcuma longa*)	13.21 ± 1.44	7.68 ± 0.56	47.72 ± 2.01	3.61	6.21
White turmeric (*Curcuma mangga* Valeton & Zijp)	39.01 ± 2.45	28.52 ± 5.53	>100 ^#^	>2.56	>3.51
Zedoary (*Curcuma zedoaria*)	34.78 ± 0.88	19.48 ± 0.21	42.26 ± 3.15 ^#^	1.22	2.17
Ginger (*Zingiber officinale*)	18.46 ± 4.23	17.07 ± 1.98	70.63 ± 2.94	3.83	4.14

^#^ Values from our previous report [23], using the same crude ethanolic extracts from Zingiberaceae plants, were used for selectivity index calculation.

**Table 2:** IC_50_ values and selectivity indices of the major active compounds from ginger in lymphoma cell lines and normal PBMCs.

Compound	IC_50_ Value (Mean ± SD)			Selectivity Index (SI)	
	Raji	Daudi	PBMCs	Raji	Daudi
6-Gingerol (µg/mL)	96.11 ± 3.16	53.79 ± 15.40	>100	>1.04	>1.86
6-Shogaol (µg/mL)	3.31 ± 0.29	2.45 ± 0.16	10.02 ± 0.96	3.02	4.09
Curcumin (µg/mL)	4.81 ± 0.99	3.59 ± 0.53	21.51 ± 0.98	4.47	5.99
Doxorubicin (ng/mL)	61.00 ± 12.86	20.79 ± 2.20	>2000	>32.79	>92.20

Data are expressed as mean ± SD from three independent experiments. SI was calculated as the ratio of IC_50_ in PBMCs to IC_50_ in cancer cells.

**Table 3:** Component-target molecular docking of shogaol against c-Myc, PI3K, Akt, Bcl2, Bax, Casp3, and Casp9.

Compound	Binding Energy (kcal/mol)						
	c-Myc	PI3K	Akt	Bcl2	Bax	Casp3	Casp9
Shogaol	−6.5	−7.0	−8.2	−6.6	−7.1	−7.0	−8.4

## Data Availability

The original contributions presented in the study are included in the article/Supplementary Material, further inquiries can be directed to the corresponding authors.

## Supplementary Materials

The following supporting information can be downloaded at: https://www.mdpi.com/article/10.3390/ijms27104168/s1.

## Abbreviations

The following abbreviations are used in this manuscript:

Casp3	Caspase-3
Casp9	Caspase-9
cl-Casp3	Cleaved caspase-3
c-Myc	Cellular myelocytomatosis
p-c-Myc	Phosphorylated c-Myc
DLBCL	Diffuse large B-cell lymphoma
GAPDH	Glyceraldehyde 3-phosphate dehydrogenase
GO	Gene Oncology
IC	Inhibitory concentration
KEGG	Kyoto Encyclopedia of Gene and Genome
mTOR	Mammalian target of rapamycin
MTT	3-(4,5-dimethythiazol-2-thizolyl)-2,5-diphenyl tetrazolium bromide
NHL	Non-Hodgkin lymphoma
PPI	Protein–protein interaction
PBMCs	Peripheral blood mononuclear cells
PI3K	Phosphoinositide 3-kinase
SI	Selective index
