# Supplementary material for: 6-Shogaol from Zingiber officinale Induces Cell Cycle Arrest via Suppression of c-Myc Protein Expression and Promotes Apoptosis in Human B-Cell Lymphoma
Source: Int J Mol Sci. 2026 May 7;27(10):4168. doi: 10.3390/ijms27104168 (PMC13207283; doi:10.3390/ijms27104168)
Supplement: Supplementary file 1 [file ijms-27-04168-s001.zip › ijms-4295204-supplementary.pdf]

Supplementary data

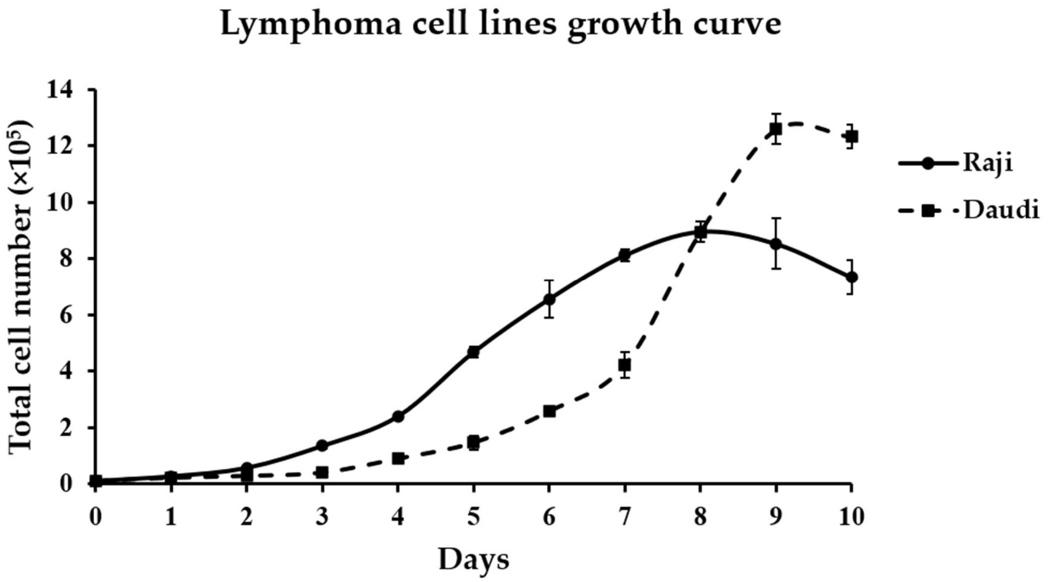

Supplementary Figure S1. Cell growth kinetics of Raji and Daudi lymphoma cell lines assessed by trypan blue exclusion assay. Viable cells were enumerated daily over a 10-day period under standard culture conditions. The solid line represents Raji cells; the dashed line represents Daudi cells.

Supplementary Table S1. List of top 20 analyzed protein-protein interaction (PPI) nodes.

Shared name	Gene Name	Degree	Averages Shortest Path Length	Clustering Coefficient	Closeness Centrality	Betweeness Centrality	Neighborhood Connectivity
AKT1	AKT1	155	1.457746479	0.221365731	0.685990338	0.082137416	42.32903226
ALB	ALB	141	1.507042254	0.216413374	0.663551402	0.092685461	41.65957447
EGFR	EGFR	128	1.556338028	0.278912402	0.642533937	0.04604443	46.484375
HSP90AA1	HSP90AA1	123	1.570422535	0.282287085	0.6367713	0.042020958	47.25203252
ESR1	ESR1	113	1.612676056	0.302939317	0.620087336	0.038248437	49.01769912
SRC	SRC	113	1.644366197	0.313685209	0.608137045	0.034818392	48.40707965
CASP3	CASP3	112	1.616197183	0.341698842	0.618736383	0.020758721	51.41071429
HSP90AB1	HSP90AB1	104	1.644366197	0.319641524	0.608137045	0.029134413	50.07692308
MMP9	MMP9	104	1.669014085	0.31982823	0.599156118	0.031672987	48.98076923
PPARG	PPARG	98	1.679577465	0.297285925	0.595387841	0.033540497	48.54081633
MTOR	MTOR	94	1.700704225	0.401967513	0.587991718	0.014332581	54.56382979
GSK3B	GSK3B	87	1.711267606	0.376369955	0.58436214	0.020973655	53.51724138
IGF1	IGF1	87	1.73943662	0.394546913	0.574898785	0.011225097	54.51724138
RHOA	RHOA	84	1.788732394	0.395582329	0.559055118	0.012432983	53
BCL2L1	BCL2L1	81	1.764084507	0.449074074	0.566866267	0.010489713	57.32098765
MAPK1	MAPK1	78	1.75	0.408591409	0.571428571	0.011817109	55.48717949
MDM2	MDM2	78	1.764084507	0.433899434	0.566866267	0.010980764	55.87179487
PIK3CA	PIK3CA	76	1.841549296	0.479649123	0.543021033	0.005102049	56.21052632
MMP2	MMP2	75	1.799295775	0.417297297	0.555772994	0.011213051	55.16
IL2	IL2	74	1.767605634	0.361347649	0.565737052	0.025459584	53.90540541
